# Analyzing the influence of manufactured sand and fly ash on concrete strength through experimental and machine learning methods

**DOI:** 10.1038/s41598-025-88923-3

**Published:** 2025-02-10

**Authors:** S Sathvik, Solomon Oyebisi, Rakesh Kumar, Pshtiwan Shakor, Olutosin Adejonwo, Adithya Tantri, V Suma

**Affiliations:** 1 Department of Civil Engineering, Dayananda Sagar College of Engineering, Bengaluru, 560111 Karnataka India; 2 Department of Civil Engineering and Geomatics, Durban University of Technology, Durban, South Africa; 3 Technical College of Engineering, Sulaimani Polytechnic University, Sulaymaniyah, Iraq; 4 Department of Civil Engineering, University of Ibadan, Ibadan, Nigeria; 5 Department of Civil Engineering, Manipal Institute of Technology Bengaluru, Manipal Academy of Higher Education, Manipal, 576 104 Karnataka India; 6 Department of Computer Science and Design, Dayananda Sagar College of Engineering, Bengaluru, 560111 Karnataka India

**Keywords:** Compressive strength, Concrete, Fly ash, Machine learning, Manufactured sand, Sustainable production, Structural materials, Techniques and instrumentation, Civil engineering, Software

## Abstract

River sand supplies are decreasing due to overexploitation and illicit sand mining. One ton of Portland cement production (the main binder in concrete) emits about one ton of carbon dioxide into the atmosphere. Thus, this study replaced conventional cement and river sand (R sand) with recycled waste materials (fly ash and manufactured sand (M sand)). The concrete mix proportions were designed using M40 grade, and the Ordinary Portland cement (OPC) and R sand were replaced with 0–85 wt% of fly ash and 0-100 wt% of M sand. The concrete samples were tested for compressive strength after 3–90 days of curing. Furthermore, machine learning (ML) techniques were engaged to predict the compressive strength of the concrete samples using Extreme Gradient Boosting (XGBoost), Long Short-Term Memory (LSTM), Support Vector Machine (SVM), and Gaussian Process Regression (GPR). Besides, the concrete samples containing fly ash, M sand, and R sand were characterized for microstructures and elemental compositions using SEM-EDS. The results revealed improved concrete compressive strength by incorporating fly ash and M sand. After 28 days of curing, OPC and R sand were partially replaced with 25 and 50 wt% of fly ash and M sand attained the designed strength of M 40 grade concrete. XGBoost model yielded the most accurate performance metrics for forecasting the compressive strength in training and testing phases with R^2^ values equal to 0.9999 and 0.9964, respectively, compared to LSTM, SVM, and GPR. Thus, the XGBoost approach can be a viable technique for forecasting the strength of concrete incorporating fly ash and M sand. SEM-EDS analyses revealed compact formations with high calcium and silicon counts. Thus, the XGBoost approach can be a viable technique for forecasting the strength of concrete incorporating fly ash and M sand.

## Introduction

Cement concrete is the most widely used building material globally, with an approximate yearly production of six billion tons, owing to its durability, flexibility, affordability, and strength^1^. Concrete ranks as the second most consumed material worldwide, closely behind water in terms of utilization. It takes about 2.8 tons of raw ingredients, including fuel and supplementary materials, to produce one ton of Ordinary Portland Cement (OPC)^2^. About one ton of carbon dioxide (CO_2_) is created for every ton of cement during the lime decarbonization process in cement manufacturing, which adds to environmental issues such as global warming^1^. About 5–8% of global carbon emissions are attributable to cement production, making it the second largest source of greenhouse gases^3^. Integrating alternative cementitious materials into cement blending can reduce greenhouse gas emissions associated with cement production. Using mineral additives, such as fly ash^4^, corncob ash^5^, cashew nutshell ash^6^, and Ground Granulated Blast Furnace Slag (GGBS)^5,7^, as cement substitutes in concrete production yields positive effects on the environment and the economy. This preserves the environment and saves a significant quantity of natural resources. Polymer-Modified Mortar (PMM) has been shown in numerous studies to be an effective material for reinforcing and repairing Reinforced Concrete (RC) structures. Although brittle failure occurred after crack reopening, Muhammad Waqas et al.^8^showed that PMM could restore up to 90% of the load-carrying capacity in shear-deficient RC beams, with medium-depth beams exhibiting the best performance. Because PMM is compatible with substrate concrete, has a higher tensile strength, and shrinks less than other repair materials, Waqas et al.^9^ found it to be superior. The study points to PMM as a dependable and reasonably priced option for RC repair and retrofitting. Waqas et al.^10^ investigated the structural performance of Geo-Polymer Concrete (GPC) columns that used Quarry Rock Dust (QRD) in place of some of the slag. Steel fibers greatly increased the reinforced GPC’s ductility, load-bearing capacity, and resistance to cracking. Its strength was 5–7% greater than that of regular concrete, indicating its potential as a sustainable substitute. The effects of bentonite and polypropylene fibers on GPC were also examined by Waqas et al.^10^, who discovered that bentonite improved workability and microstructural integrity while polypropylene fibers increased tensile and flexural strength. These investigations support previous studies on PMM, emphasizing the vital role that material compatibility and creative additives play in enhancing the robustness, sustainability, and longevity of concrete structures^4–6^.

The studies discussed above emphasize the importance of adopting sustainable materials like PMM for repairing and retrofitting RC structures. Similarly, thermal power plants generate Fly Ash (FA), a byproduct of burning coal, which poses significant environmental challenges when dumped in open spaces, affecting soil fertility. Fly ash, composed of silica or silica plus alumina, reacts with calcium hydroxide and generates cementitious characteristics. This interplay substantially affects the properties of freshly mixed and hardened concrete^11,12^. Adding FA decreases the permeability of concrete and lowers the early aging of the concrete. Using sulfate-resistant cement in concrete with a low water-to-cement ratio is advisable to minimize sulfate attack^13,14^. Because of pozzolanic reactions, there is less calcium hydroxide present in cement-based concrete, which increases the concrete’s resistance to harsh exposure conditions. For instance, cement can be replaced with 15–20 wt% of FA, and the results can exhibit better concrete mixes. These concrete mixes have inherent qualities that increase durability, resist sulfate attack, alkali-silica reaction, and lower chloride penetration^15^. Aggregates, acting as a low-cost filler in concrete and mortar, are essential to the mechanical strength, durability, stiffness, shrinkage, insulation, and high-temperature performance of concrete. Usually, 70–80% of the volume of concrete is made up of coarse and fine aggregates, with 35–45% comingfrom fine aggregates^16,17^. Robust, durable, and cost-effective concrete requires high-quality fine aggregates. Although natural or river sand is commonly utilized because of its advantageous qualities, overuse of it harms the environment. It can cause harm to water bodies and groundwater depletion^17^. Alternatives such as M-sand or crushed quarry rock should be considered a solution. They provide a sustainable and environmentally responsible way to lessen dependency on natural sand^18–20^.

From the perspective of the literature, a study substituted M sand (waste foundry sand) and FA for natural sand and cement in the manufacturing of Steel Fibre Reinforced Concrete (SFRC)^19^. The results indicated that at 34 and 20% of M sand and FA, the mechanical characteristics of SFRC were optimal. Compared to the control concrete, the compressive, split tensile, and flexural strengths rose by 28.8, 33.5, and 33.8%. According to microanalysis, concrete’s porosity can be decreased, and its advantageous pores can be increased with moderate M sand and FA. Conversely, replacing natural sand and cement with 60% and 30% of M sand and FA can impede the hydration process and raise the concrete’s porosity^19^. The results of the experimental investigation’s tests on roller-compacted concrete pavement containing FA and M sand showed that, at all FA replacement levels, replacing 50% of the M sand with river sand produced higher compressive strength, ultrasonic pulse velocity, and dynamic modulus of elasticity. However, 10 wt% of FA combined with 90 wt% of cement showed the best results^20^. This is because the aggregate was adequately packed, raising the packing density of concrete^20–28^. The factional replacement of M sand with granulated blast furnace slag (GBFS) at different intervals was investigated. Concrete with 60% GBFS and 40% M sand as fne aggregates showed the best mechanical strengths. Compared to the control sample, the durability results showed better acidic and sulfate resistance at the same replacement level^16^. The effects of replacing fine aggregates and cement with M sand (foundry sand, FS) and FA on the mechanical properties of concrete were investigated. The results indicated that 30 and 60% of FA and M snd substitutes attained better mechanical properties^21^. The mechanical, durability, and sustainable studies of fly-ash-based lateralized concrete incorporating M sand were examined. The residual compressive strength of concrete was significantly increased by using a mixture of 75% M sand and 25%laterite scrapsas fine aggregates. After 28 days, this combination increased compressive strength by around 11%^22^.

Furthermore, laterized concrete mixes, including 50% M sand and 50% laterite scrap substitutes, showed acceptable limits for durability characteristics. The assessment of embodied energy of laterized concrete mixes attained a significant decrease of 22.41 MJ/m^3^^22^. The effects of replacing natural river sand with geopolymer fly ash sand on the hardened properties of concrete were investigated by a previous study^23^. Comparable mechanical strength was observed between the geopolymer fly ash sand concrete and the river sand concrete. In addition, using geopolymer fly ash sand in the concrete improves its durability^23^. The manufacturing of High-Flowability Concrete (HFC) can effectively substitute natural sand to the tune of 40% superfine river sand and 60% coarse M sand. Furthermore, it is possible to admix a significant amount of FA at a 45 binder’s content without compromising the development and strength of HFC. In addition, HFC with a 75% FA content can be produced, maintaining a comparable strength development to conventional concrete mixed FA^24^. The mechanical characteristics and post-fire performance of alkali-activated-FA-based concrete incorporating M sand (waste from the granite industry) and polypropylene fibres were studied. After 30 min of fire exposure, the residual compressive strengths of concrete at 25% of M and substitution were reduced to a range of only 25–39%^25^. The effects of FA and GGBFS as cement substitutes and M sand as sand replacement materials on concrete strength were investigated. The replacement levels at 23% FA, 47% GGBFS, and 25% FS and recycled concrete aggregates attained a higher compressive strength than conventional concrete^26^.

As mentioned earlier, the studies provided significant insights into utilizing FA and M sand in concrete production. However, researchers have not yet established models for forecasting the FA-based concrete incorporating M sand using machine learning (ML) techniques. For this reason, developing a prediction model for FA-based concrete that incorporates M sand based on ML algorithms is crucial for optimizing and producing concrete. Creating explicit models for blended concrete using experimental data frequently results in low accuracy and limited generalization capacity^27^. There are three primary causes for these limitations. First, the grade of fly ash is contingent upon the quality of coal utilized and the operational state of the thermal power plant. Second, many factors affect FA-M sand-based concrete; including them in an explicit model can be challenging. Third, creating an explicit model for every factor limits the model’s ability to be generalized, rendering it unsuitable for engineering applications. Thus, developing a high degree of generality and precision for the FA-M sand-based concrete prediction model is crucial for engineering applications. Due to scientific and technological advancements, machine learning offers a unique method for creating concrete models. One of the main areas of artificial intelligence is ML, which focuses on applying mathematical models and algorithms to make computers capable of performing particular tasks without the need for explicit programming instructions^28–30^. Making predictions or making decisions by analyzing large amounts of data and identifying hidden patterns or rules is the core concept of ML. Machine learning is commonly used in engineering for a variety of tasks, including the forecast of strength^31–33,34^ structural health monitoring^35^, cancer risks^36^, radioactive hazards^37–39^ concrete mix design^40^, transport analytics^41^, durability^42,43^, and shrinkage and creep^44,45^. Engineers can increase structural safety and extend the life of structures by using ML techniques to make highly accurate decisions based on historical data and on-site observations. The accuracy and dependability of FA-M sand-based concrete models are ensured by ML approaches, providing a fresh viewpoint to overcome the drawbacks of traditional models.

To forecast the compressive and flexural strengths of environmentally friendly jarosite-mixed concrete, Gupta and Kumar^11^ experimented with various machine learning models. The Feed-Through Elman Neural Network (FTENN), which they proposed, performed better than more conventional models like, Elman Neural Network (ENN), Feed-Forward Neural Network (FFNN), Diagonal Recurrent Neural Network (DRNN), and Jordan Recurrent Neural Network (JRNN). With RMSEs of 0.021 (compressive) and 0.041 (flexural) during training and 0.029 (compressive) and 0.052 (flexural) during testing, the FTENN model produced the lowest errors. Additionally, it demonstrated superior prediction accuracy and generalization by recording the lowest MAPE values for compressive and flexural strengths, respectively, at 6.35% and 14.60%. Advanced models like XGBoost provide significant advantages over traditional models like FTENN due to their ability to handle complex, non-linear relationships and efficiently optimize performance. These modern techniques are better suited for diverse and sustainable applications in concrete research.

This research aims to substitute OPC and river sand (R sand) with fly ash (FA) and manufactured sand (M sand), made from waste ceramic tile aggregates, at 0–85 wt% and 0–100 wt% replacement levels. The concrete mixes were proportioned using M40 grade, and the compressive strengths were tested after 3–90 days. By substituting fly ash and manufactured sand (M sand) for traditional materials, this project tackles the depletion of river sand and the elevated carbon emissions from cement production. The goal of the project is to increase the strength of concrete while lowering resource consumption and the environmental impact of concrete production. The construction industry will find this research especially helpful as it provides sustainable substitutes for high-strength concrete used in infrastructure projects. By decreasing dependence on natural resources and optimizing mix designs through machine learning for accurate strength prediction, this work offers a cost-effective and environmentally friendly way to build long-lasting structures.

The utility of Machine Learning (ML) models such as Extreme Gradient Boosting (XGBoost), Long Short-Term Memory (LSTM), Support Vector Machine (SVM), and Gaussian Process Regression (GPR) lies in their ability to predict compressive strength with high accuracy. These models enable efficient optimization of mix proportions, reducing the need for extensive trial-and-error testing. Additionally, these ML techniques can help identify the most influential factors affecting strength and ensure consistent quality in concrete production. Based on experimental data results, the concrete samples were also characterized for microstructures and elemental compositions using scanning electron microscopy equipped with energy dispersive spectroscopy (SEM-EDS). Overall, this research contributes to optimizing mix proportions for producing FA-M sand-based concrete, resulting in time and energy savings, as well as improved quality and performance.

## Methodology

**Fig. 1:**
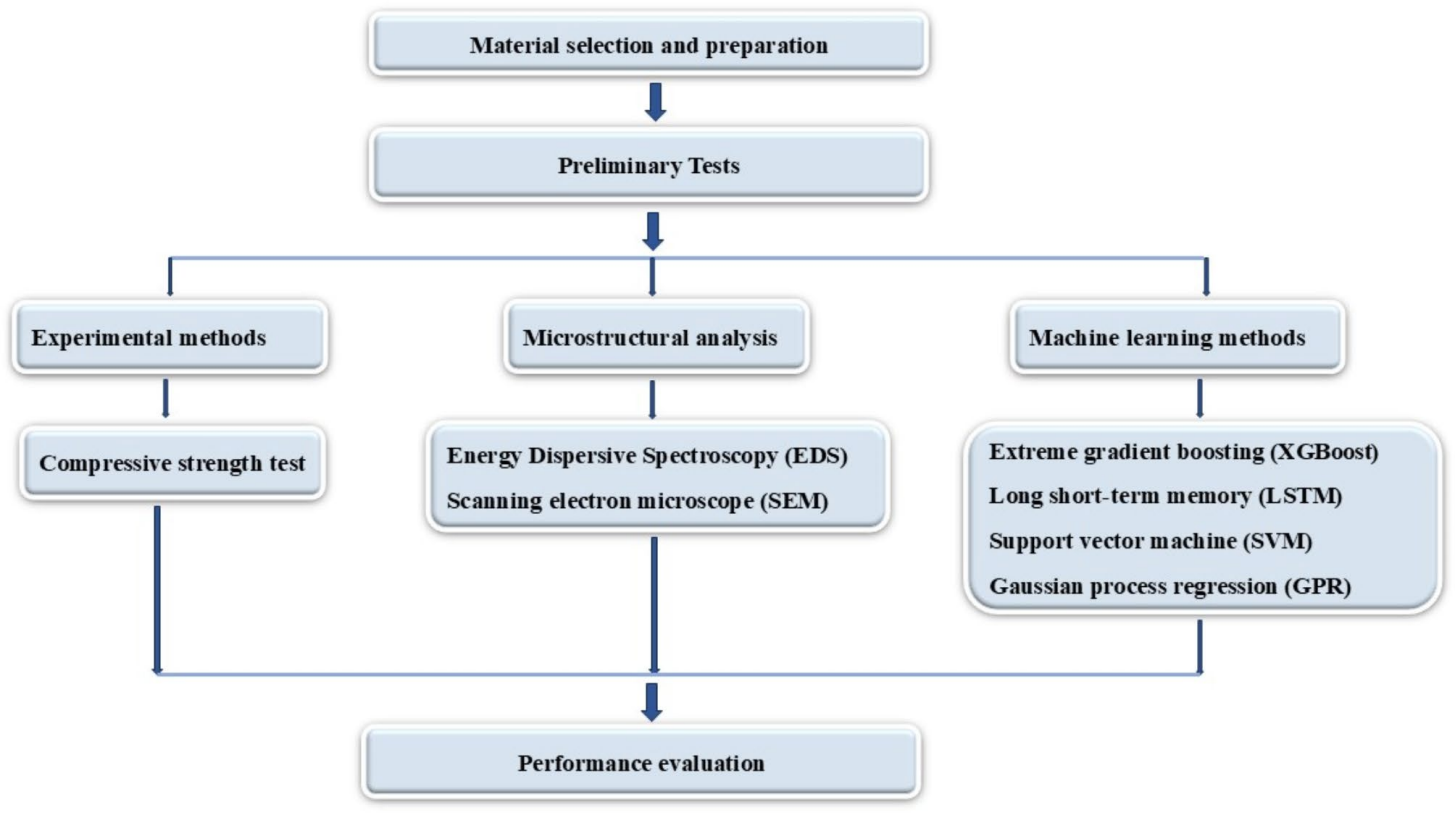
Methodology.

The methodology adopted in this study is presented in a flow diagram in Fig. 1. The study substitutes Ordinary Portland Cement (OPC) and River Sand (R Sand) with Fly Ash (FA) and Manufactured Sand (M Sand) in M40-grade concrete. Compressive strength was evaluated at 3, 7, 28, and 90 days, with microstructural analysis using Scanning Electron Microscopy (SEM) and Energy Dispersive Spectroscopy (EDS). Machine learning models, including XGBoost, LSTM, SVM, and GPR, were developed to predict compressive strength from experimental data, validated through statistical metrics and visual tools, emphasizing optimal material substitution for sustainable concrete production.

## Materials and methods

### Materials

**Fig. 2:**
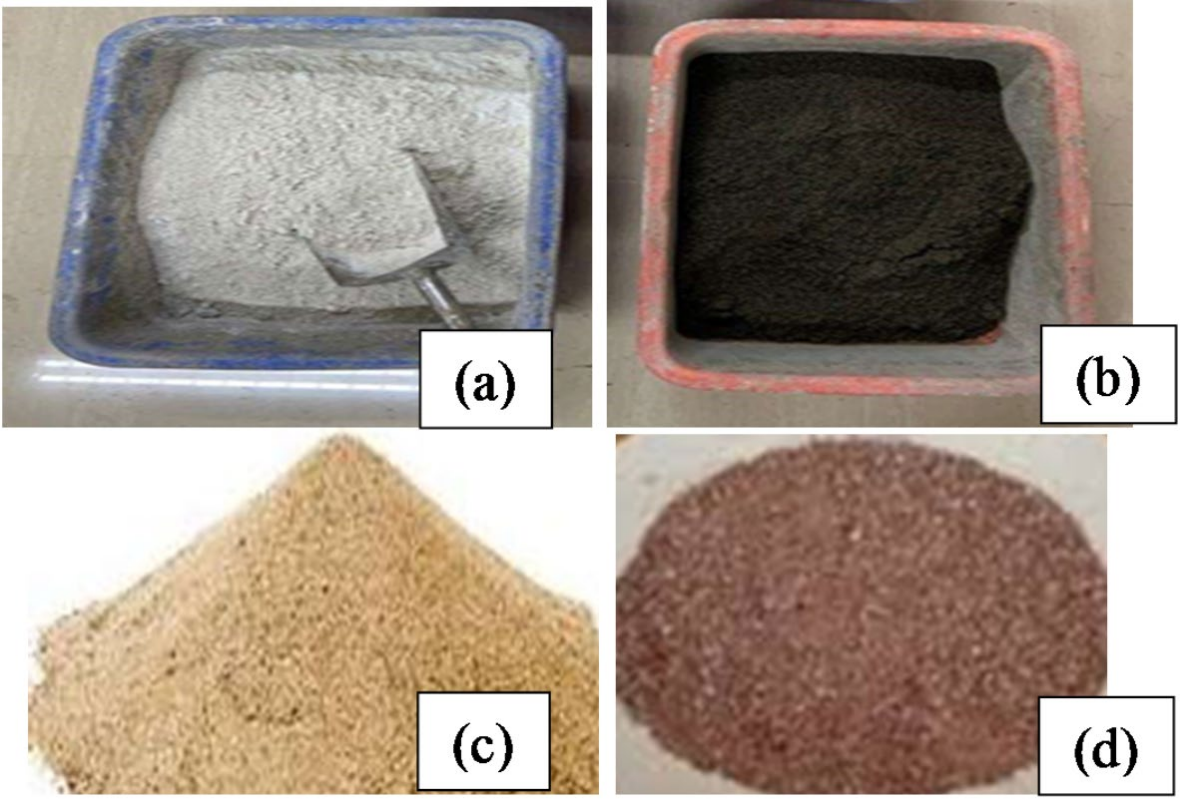
Materials used (**a**) OPC, (**b**) fly ash, (**c**) R sand, and (**d**) M sand.

A 53-grade ordinary Portland cement (OPC) adhering to the Indian Standard (IS)^46^ was used. Fly ash obtained from Raichur thermal power station, Shaktinagar, Karnataka, India, was used as an OPC substitute. According to IS, the physical characteristics, such as bulk density (BD) and specific gravity (SG)^47^, fineness^48^, consistency^49^, and initial setting time (IST) and final setting time (FST)^50^ of the fly ash and OPC depicted in Fig. 2 were ascertained. The results are presented in Table 1. From Table 1, OPC’s SG, consistency, IST, and FST met the IS recommendations of 3.10–3.15, 30–35%, 30 mn minimum, and 600 min maximum^46^. Figure 2 shows the fine aggregates (R sand and M sand) used.

**Table 1:** Properties of OPC and fly ash.

Property	OPC	Fly ash
Colour	Grey	Pale grey
SG	3.12	1.92
BD, kg/m^3^	1131	1163
Fineness, m^2^/kg	237	269
IST, min	41	-
FST, min	114	-
Consistency, %	31	-

Manufactured sand (M sand) was sourced from Ramanagaram quarry, Bangalore, India. M sand was used as a substitute for the natural river sand (R sand). The M sand (waste ceramic tile aggregate) and R sand were fine aggregates. The physical properties of M and R sands were evaluated following IS standard^51^, and the results are shown in Table 2. Aggregates passing through a 20 mm sieve and retained on an IS sieve of 10 mm were used as coarse aggregates. The SG, Fineness Modulus (FM), Moisture Content (MC), BD, and Water Absorption (WA) of fine aggregates satisfied the IS specifications of 2.5–2.9, 3.2 max., 2% max., about 1750 kg/m^3^, and 2–4%^52^. Besides, M and R sands fell within Zone 2 per IS standard^53^.

**Table 2:** Properties of the fine aggregates.

Property	*R* sand	M sand
SG	2.65	2.67
FM	2.60	2.90
MC, %	0.42	0.35
WA, %	1.22	1.12
Loose BD, kg/m^3^	1352.54	1690.84
Compacted BD, kg/m^3^	1505.66	1774.71
Zone	2	2

## Experimental methods

The concrete mix was designed with M40 structural concrete grade following the IS^54^. During mix designs, physical properties such as SG, WA, and MC of constituents were considered. Based on IS guidelines^54^, OPC, fly ash, M sand, and R sand ranged between 48.78 and 295 kg/m^3^, 0 and 246.22 kg/m^3^, 0 and 801 kg/m^3^, 0 and 801 kg/m^3^.

The compressive strength was tested following IS code book^55^ on cube sizes 100 mm × 100 mm × 100 mm using a computerized compression testing machine with a 2000 kN maximum capacity and a 2.3 kN/s rate of force. The concrete samples were removed from the moulds after 24 h, submerged in water, and tested at 3, 7, 28, and 90 days.

### Microstructural analysis

The concrete samples were subjected to Energy Dispersive Spectroscopy (EDS) with a Scanning Electron Microscope (SEM) to examine what factors led to the concrete’s improved qualities. The primary purpose of these analytical methods was to identify and classify the elements contained in the concrete^56^. The microstructural characterization involved the analysis of powdered samples with particle sizes less than 90 microns. After 28 days of curing, hardened concrete specimens were crushed to obtain the powdered samples. The MINI-SEM SNE-3200 M instrument was engaged to conduct SEM and EDS studies, providing a resolution as low as 50µ^57^. This helps image the concrete microstructure and analyze its elements^58–60^.

## Machine learning methodology

### Extreme gradient boosting (XGBoost)

The XGBoost algorithm was created by Chen and Guestrin^60^ and, because of its exceptional accuracy^61^, has been widely used in numerous fields. XGBoost has become a strong competitor in the ever-changing field of machine learning, drawing interest from practitioners and researchers due to its remarkable capacity to simulate complex processes in various domains. This algorithm, which combines gradient-boosting decision trees, is an example of the effectiveness of ensemble approaches in predictive modeling. Fundamentally, XGBoost uses the combined knowledge of several decision trees, understanding that even though a single tree may perform poorly, the combined effect of these trees produces unmatched predictive accuracy. This essence highlights the basic idea behind boosting algorithms: combining several weak learners creates a robust predictive model. Notably, the second-order Taylor’s series function is used in the XGBoost approach to expand the loss function, and a regulation term is added to address the overfitting problem^44,62^. Equation 1 represents the objective function that XGBoost implements with $\:{y}_{i}$ and $\:{\widehat{y}}_{i}$ representing the actual and predicted values, respectively. The difference between the expected and actual values is represented by $\:{\sum\:}_{i}l\left({\widehat{y}}_{i},{y}_{i}\right)$, where $\:l$ is the difference loss function.

1 $$\:\mathcal{L}={\sum\:}_{i}l\left({\widehat{y}}_{i},{y}_{i}\right)+{\sum\:}_{k}{\Omega\:}\left({f}_{k}\right)$$

2 $$\:{\Omega\:}\left({f}_{k}\right)=\gamma\:T+0.5\lambda\:{\Vert w \Vert}^{2}$$

The loss function is defined in this study using the mean square error (MSE). $\:{f}_{k}$ is the model of the kth tree, and $\:{\Omega\:}$ is a regular penalty function (Eq. 2). The term $\:{\sum\:}_{k}{\Omega\:}\left({f}_{k}\right)$, which represents the model’s generalization and ultimately optimizes the model’s complexity^63^. Two factors influence the accuracy of an XGBoost model: (i) the total number of estimators or model trees. Tree depth, or the distance from the root to the output leaf, measures model complexity; (ii) higher estimators should be considered for more complicated models. Although a deep tree can improve the model’s accuracy, it can also cause overfitting, so it is essential to choose an optimized depth.

### Long short-term memory (LSTM)

Hochreiter and Schmidhuber introduced the LSTM network in 1997^64,65^ to address the issues of “gradient disappearance” and “gradient explosion” by implementing the gating function mechanism. The LSTM recurrent neural network model can effectively extract features from time-series data by extracting both the long-term and short-term dependencies of the data. The principal structure of the LSTM consists of a forget gate, an input gate, an update gate, and an output gate^66^. Equations 3-8 are the primary formulas for the LSTM structure, where $\:{W}_{f}$, $\:{W}_{i}$, $\:{W}_{g}$, and $\:{W}_{o}$ are weight vectors; $\:{b}_{f}$, $\:{b}_{i}$, $\:{b}_{g}$, and $\:{b}_{o}$ are bias vectors; $\:{c}_{t}$ and $\:\sigma\:$ are memory cell and sigmoid activation functions, and $\:{f}_{t}$, $\:{i}_{t}$, $\:{g}_{t}$, and $\:{o}_{t}$ determine the output values of the forget, input, update, and output gates.

3 $$\:{f}_{t}=\sigma\:({W}_{f}\left({h}_{t-1},{x}_{t}\right)+{b}_{f})$$

4 $$\:{i}_{t}=\:\sigma\:({W}_{i}\left({h}_{t-1},{x}_{t}\right)+{b}_{i})$$

5 $$\:{g}_{t}=\text{t}\text{a}\text{n}\text{h}({W}_{g}\left({h}_{t-1},{x}_{t}\right)+{b}_{g})$$

6 $$\:{c}_{t}=\:{f}_{t}{c}_{t-1}+{i}_{t}{g}_{t}$$

7 $$\:{o}_{t}=\:\sigma\:({W}_{o}\left({h}_{t-1},{x}_{t}\right)+{b}_{o})$$

8 $$\:{h}_{t}={o}_{t}\text{t}\text{a}\text{n}\text{h}\left({c}_{t}\right)$$

## Support vector machine (SVM)

Classification, regression, and outlier detection are three applications of SVMs, which are supervised learning techniques. SVM is a practical algorithm for statistical learning that applies the structural risk minimization principle to constrained optimization problems, resulting in an optimal solution. SVM was first created at AT&T Bell Laboratories by Cortes and Vapnik^67^. It was used in linear and nonlinear classifications and based on statistical learning frameworks, specifically the Vapnik–Chervonenkis theory^68^. The nonlinear SVM employs kernels for classifications, while the linear SVM uses a maximum-margin hyperplane (hard- or soft-margin). Support vector clustering (SVC) is another term for SVM’s application in unsupervised learning^69^. The most popular kernel functions used by SVM algorithms are the sigmoid, polynomial, radial basis function, linear, nonlinear, and polynomial functions. As an activation function for neurons, this kernel function is comparable to a two-layer neural network perceptron model. This function can be used by modifying the prediction process in a manner akin to neural networks. Assume a training dataset denoted by$\:\:{\left\{({x}_{i},{y}_{i})\right\}}_{i=1}^{n}$, where $\:{x}_{i}\:$ and$\:{y}_{i}$ represent the input vector and corresponding output, respectively. The objective of the SVM regression model is to identify a function $\:f\left(x\right)$ that exhibits a maximum deviation of $\:\epsilon$ from each target $\:{y}_{i}$ across all training data. Equation 9 represents the estimating function, where $\:w$ represents the weight vector, $\:b$ signifies the bias term, and $\:\psi\:\left(x\right)$ represents a collection of nonlinear transformations.

9 $$\:f\left(x\right)={w}^{T}\psi\:\left(x\right)+b$$

To estimate the coefficients w and b, the regularized risk function given in Eq. 10, dependent on Eqs. 11, 12, and 13, is minimized.

10 $$\:\frac{1}{2}{w}^{T}w+C\sum\:_{i=1}^{n}{\xi\:}_{i}+C\sum\:_{i=1}^{n}{\xi\:}_{i}^{*}$$

11 $$\:{w}^{T}\psi\:\left({x}_{i}\right)+b-{y}_{i}\le\:\epsilon+{\xi\:}_{i}^{*}$$

12 $$\:{y}_{i}-{w}^{T}\psi\:\left({x}_{i}\right)-b\le\:\epsilon+{\xi\:}_{i}$$

13 $$\:{\xi\:}_{i},\:{\xi\:}_{i}^{*}\ge\:0,\:i=1,\:2,\:\dots\:,\:n$$

## Gaussian process regression (GPR)

An example of a nonparametric probabilistic kernel model is GPR^70^. This machine learning technique has become increasingly popular in the literature in the last few years. This technique is not limited to prediction alone; it can also yield the confidence interval for every prediction point, thereby quantifying the forecast’s uncertainty. A group of random variables with a shared Gaussian distribution for every variable is known as a GPR^71^. A Gaussian process essentially expands the corresponding probability distribution. With an input vector as input, the Gaussian distribution calculates the probability of the vector’s mean and variance. The likelihood of a time series vector input is computed for each time step^72^. Thus, instead of computing a mean and variance, the GPR model calculates a mean and covariance vector, which are scalar variables. Equation 14 can be used to formulate the GPR model with Gaussian noise, where $\:x$ and $\:y$ represent the input and output vectors, respectively. The regression function $\:f\left(x\right)$ is used. $\:\epsilon\:$ is the noise term, which has a mean of 0 and a standard deviation of $\:{\sigma\:}_{n}$ and is typically assumed to follow the Gaussian distribution $\:({\epsilon}\sim{N}(0,{\sigma\:}_{n}^{2})$.

14 $$\:y=f\left(x\right)+\epsilon$$

Equation 15 states that a mean $\:m\left(x\right)$ and a covariance $\:k\left(x,\:{x}^{{\prime\:}}\right)$ can fully characterize a function $\:f\left(x\right)$. Typically, $\:m\left(x\right)$ is set to 0 for computational and notational convenience. The covariance function, also referred to as the kernel function, is selectable.

15 $$\:f\left(x\right)\sim{GP}\left(m\left(x\right),k\left(x,\:{x}^{{\prime\:}}\right)\right)$$

### Performance evaluation

Here, the accuracy domain was explored by investigating four powerful models (XGBoost, LSTM, SVM, and GPR) that attempt to predict the concrete compression strength with a high degree of certainty. A wide range of performance metrics was determined to evaluate the accuracy and reliability of these ML models and scrutinize them all. Equations (16)-(23) reveal and derive these performance parameters, where ‘$\:k$’ denotes the total number of observations for each parameter and ‘$\:q$’ represents the total number of inputs for prediction^44,62,72–74^. The model accuracy was further explored by differentiating between the predicted outputs ($\:{\widehat{x}}_{i}$) and the actual outputs ($\:{x}_{i}$), with $\:{x}_{mean}$ encapsulating the average value derived from the input data. Understanding the predictive power and robustness of these models is highlighted by this thorough evaluation, which aims to help with informed decisions regarding concrete strength forecasting.

16 $$\:{R}^{2}=\frac{{\sum\:}_{i=1}^{k}{({x}_{i}-{x}_{mean})}^{2}-{\sum\:}_{i=1}^{k}{({x}_{i}-{\widehat{x}}_{i})}^{2}}{{\sum\:}_{i=1}^{k}{({x}_{i}-{x}_{mean})}^{2}}$$

17 $$\:WMAPE=\frac{\sum\:_{i=1}^{k}\left|\frac{{x}_{i}-{\widehat{x}}_{i}}{{x}_{i}}\right|\times\:{x}_{i}}{\sum\:_{i=1}^{k}{x}_{i}}$$

18 $$\:VAF=1-\frac{var({x}_{i}-{\widehat{x}}_{i})}{var\left({x}_{i}\right)}\times\:100\%$$

19 $$\:LMI=1-\left[\frac{\sum\:_{i=1}^{k}\left|{x}_{i}-{\widehat{x}}_{i}\right|}{\sum\:_{i=1}^{k}\left|{x}_{i}-{x}_{mean}\right|}\right],\:0<LMI\le\:1$$

20 $$\:RSR=\frac{RMSE}{\sqrt{\left[\frac{1}{k}\sum\:_{i=1}^{k}{\left({x}_{i}-{x}_{mean}\right)}^{2}\right]}}$$

21 $$\:MAE=\frac{1}{k}\sum\:_{i=1}^{k}\left|({\widehat{x}}_{i}-{x}_{i})\right|$$

22 $$\:RMSE=\sqrt{\frac{1}{k}\sum\:_{i=1}^{k}{({x}_{i}-{\widehat{x}}_{i})}^{2}}$$

23 $$\:NS=1-\:\frac{\sum\:_{i=1}^{k}{\left({x}_{i}-{\widehat{x}}_{i}\right)}^{2}}{\sum\:_{i=1}^{n}{\left({x}_{i}-{x}_{mean}\right)}^{2}}$$

#### Descriptive statistics and data preprocessing

The effectiveness of predictive models is contingent on the quality and quantity of the data utilized during their training process. In this research, datasets were curated, comprising 940 experimental data points collected with extreme care and aimed explicitly at predicting compressive strength. Four supervised ML models were constructed using this foundational dataset as the basis: XGBoost, LSTM, SVM, and GPR. At the core of the model development were six critical input parameters - age, cement, fly ash, R sand, M sand, and water; each of which significantly impacted the anticipated results. Upon further examination of these parameters, the analysis revealed an extensive assortment of crucial statistics and distributions, as detailed in Table 3. There are two fundamental classifications of descriptive statistics: variability indices and central tendency measures. The former, which includes measures such as the median, skewness, mean, and kurtosis, provides insight into the symmetry and central tendency of the dataset. In contrast, the latter, which comprises statistical measures such as variance, range, and standard deviation, provides profound insights into the variability of data points. Upon closer inspection, it was discovered that each of the six input parameters deviates minimally from a normal distribution, as indicated by the nominal skewness and kurtosis values in Table 3. The statistical robustness of the dataset enhances the credibility of subsequent analyses by imbuing it with an air of stability and dependability.

**Fig. 3:**
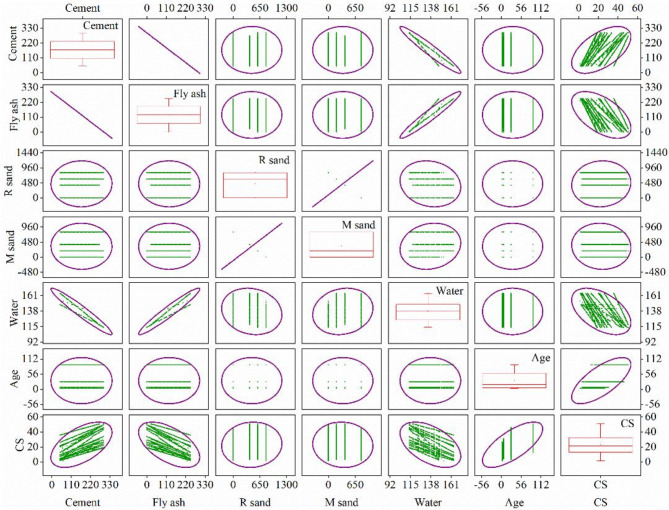
Scatter matrix of the input and output parameters.

**Fig. 4:**
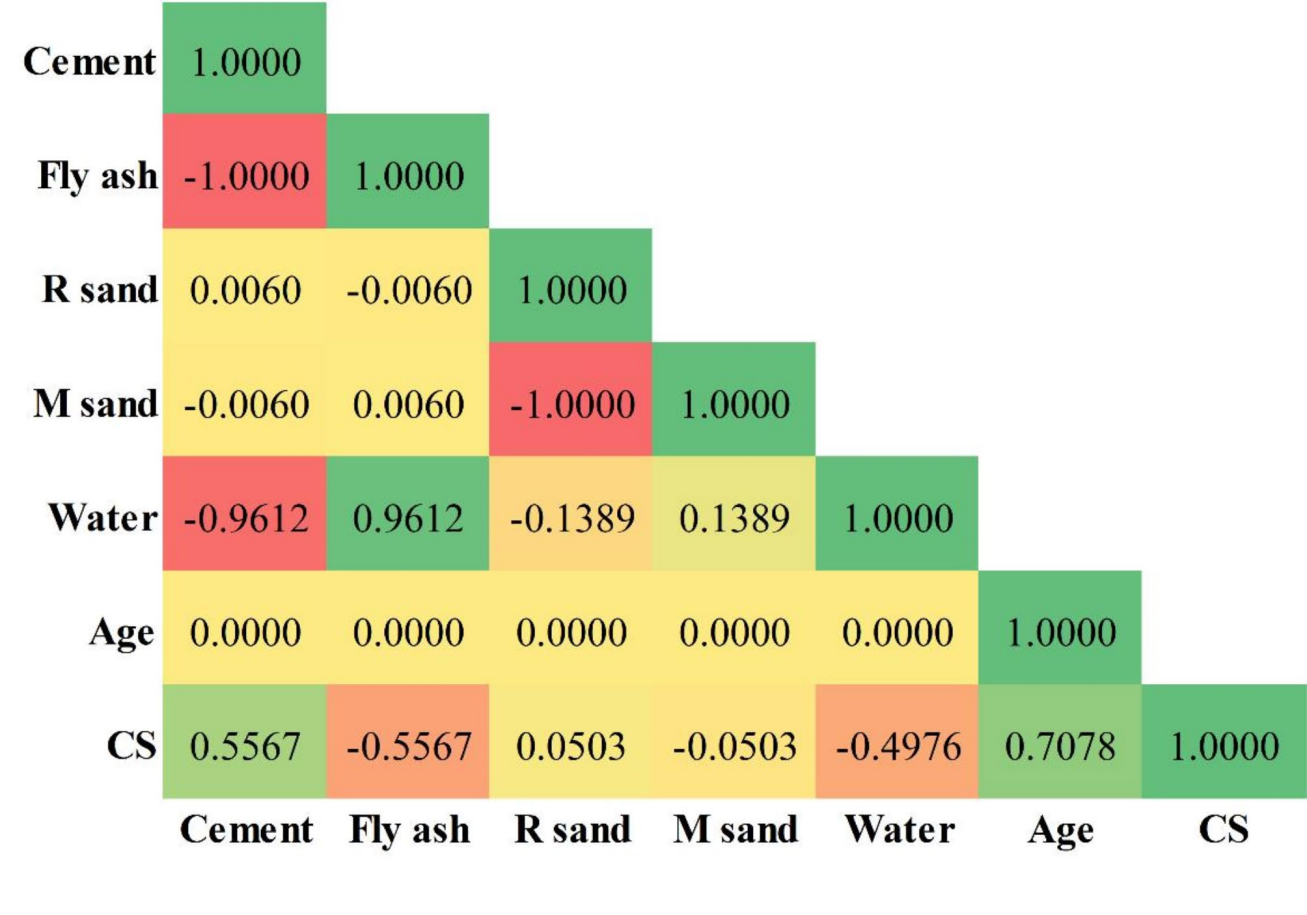
Pearson correlation matrix of the input and output parameters.

Figure 3 presents a pair plot that depicts the subtle connections between every pair of parameters to provide additional insight into the complex interactions among variables. The extent of correlation between the input and output parameters was evaluated using the Pearson correlation coefficient. The results of this effort are succinctly illustrated in Fig. 4, where distinct patterns become apparent. It is worth mentioning that fly ash (−0.5567), M sand (−0.0503), and water (−0.4976) exhibit a negative correlation with compressive strength. Conversely, cement (0.5567), R sand (0.0503), and age (0.7078) demonstrate positive correlations in this regard. These disclosures provide a significant understanding of the complex network of elements comprising concrete strength characteristics, highlighting the diverse connections between input parameters and output predictions.

**Table 3:** Descriptive statistics of the input and output parameters.

Material	Unit	Mean	Median	Standard Deviation	Minimum	Maximum	Skewness	Kurtosis
Cement	kg/m^3^	168.59	168.96	70.61	48.78	295	0.014	−1.19
Fly ash	kg/m^3^	126.41	126.04	70.61	0	246.22	−0.014	−1.19
R sand	kg/m^3^	451.63	600.75	299.38	0	801	−0.44	−1.18
M sand	kg/m^3^	349.37	200.25	299.38	0	801	0.44	−1.18
Water	kg/m^3^	137.60	138.26	14.03	114	164.05	0.11	−1.08
Age	days	32	17.5	34.83	3	90	0.92	−0.88
CS	MPa	22.99	21.18	12.31	1.53	50.67	0.38	−0.87

## Results and discussion

### Compressive strength

**Fig. 5:**
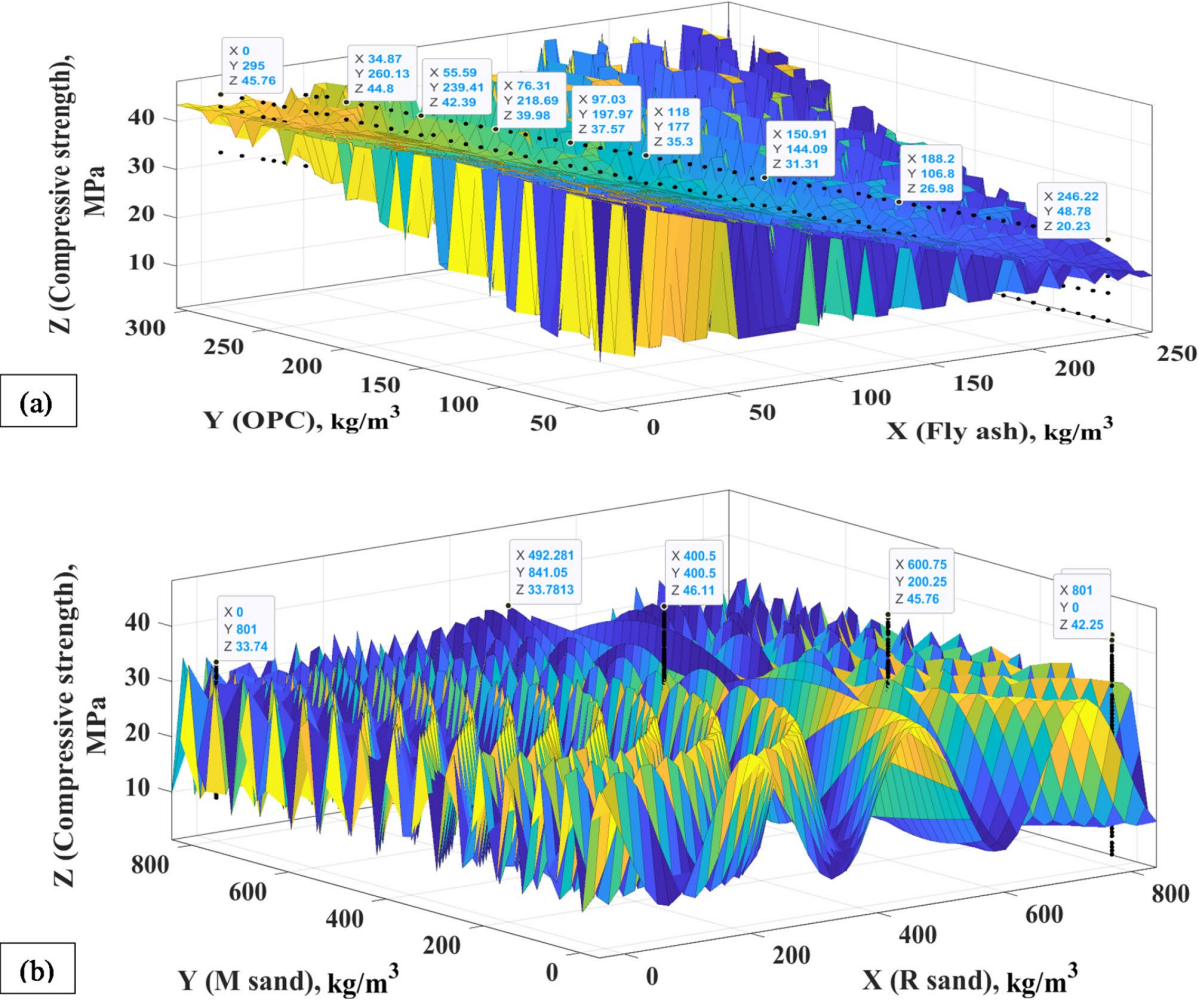
Relationship between (**a**) binders (OPC and fly ash) and compressive strength, and (**b**) aggregates (R sand and M sand) and compressive strength.

Figure 5 shows the relationship between the concrete’s constituents and compressive strength at 28 days. Figure 4(a) illustrates that the percentage replacement of fly ash was proportionally inversed to the compressive strength. At 0–25 wt% replacement of OPC with fly ash, there was about 0–13% decrease in compressive strength. Concrete’s compressive strength at 25 wt% fly ash replacement was approximately 40 MPa, which can be regarded as an optimal replacement as strength declines below the intended strength at higher replacement levels. This suggests that the smooth surface of spherical fly ash particles reduced the flow friction of cement grains and that diluting fly ash with less OPC slowed the cement’s hydration process. As fly ash reacts with the cement hydration product Ca(OH)_2_, it happens relatively early in the process and establishes the duration of the binder paste setting^75^. This suggests using a high fly ash percentage instead of cement above 25 wt% retards the hydration rate of OPC.

Moreover, the calcium silicate hydrate (C-S-H) gel results from the long-term hydration process of fly ash reacting with the hydration product Ca(OH)_2_^76^. As the amount of fly ash increases, cement hydration is slowed by the rise in temperature, a higher water-to-binder (w/b) ratio, and less cement content. This requires the cement to hydrate over an extended period to generate strength^75,77,78^. However, when fly ash is adequately hydrated, a more significant increase in strength can be obtained^79^. The maximum fly ash content for reinforced concrete structures is 35–40% in concrete made with OPC when the w/b ratio is less than 0.40. If the w/b ratio exceeds 0.40, the fly ash content should be decreased by 5%^80^. Increasing the concrete’s compactness can be achieved by filling the small grains with excess fly ash. Therefore, the compressive strength of concrete can approach the target at the typical curing age of 28 days by employing the extra fly ash to lower the w/b ratio. Ultimately, this research establishes a 25% optimum repacement of OPC with fly ash in fly ash-M sand-based concrete production, meeting the designed strength at 28 days.

Figure 5(b) signifies the relationship between the aggregate substitutes and compressive strength. The compressive strength increased with increasing M sand content and replacing R sand with 0–50 wt% M sand increases the concrete’s compressive strength from 33.74 to 46.11 MPa, indicating a 27% increment compared to conventional concrete incorporating only R sand. However, at greater replacement levels, compressive strength falls short of the designed strength, indicating a 50 wt% M sand exhibited an optimum replacement level. These findings imply that the M sand’s angular shape and rough surface texture enhanced the cement matrix’s bond with the aggregate particles and promoted aggregate interlock, increasing the concrete’s compressive strength^81^. The rough surface of M sand creates a robust mechanical force between its particles, enhancing the strength of M sand concrete with the same mix ratio^82^. According to a related study, adding M sand to concrete increased its strength, and this effect became more noticeable when M sand substitution with natural sand exceeded 40 wt%^83^. Thus, the findings show that M sands can effectively substitute R sands in cement-based concrete incorporating fly ash.

### Microstructural characteristics

**Fig. 6:**
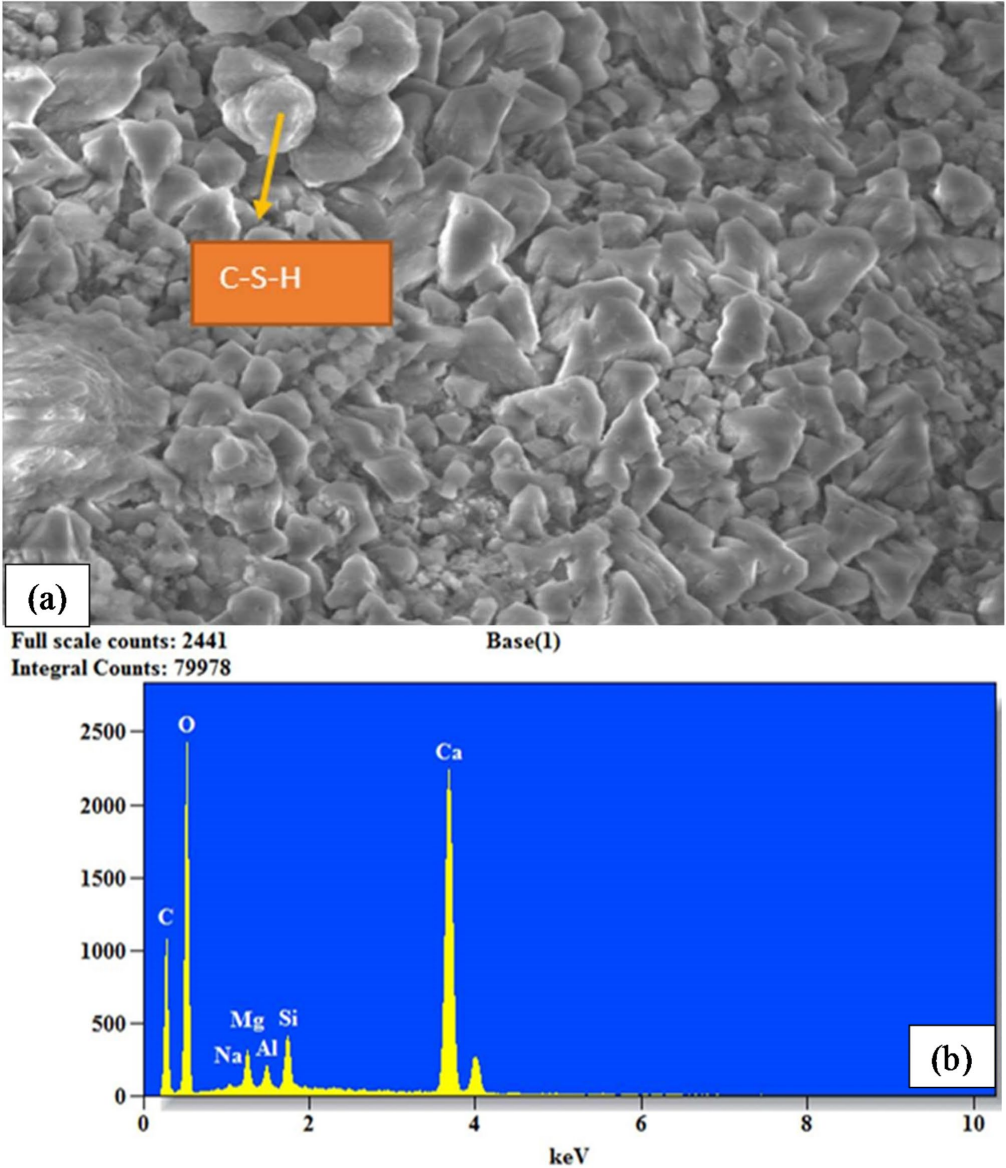
Microstructural analysis of concrete (**a**) SEM micrograph for 10% M sand and 10% R sand (**b**) EDS for 10% M sand and 10% R sand.

Figure 6 (a) and (b) show SEM micrographs and elemental compositions of concrete containing 10% R sand and 10% M sand. From Fig. 6 (a), the blend of M sand and R sand in the concrete mix resulted in denser and compact microstructures between the cementitious materials and the aggregates, generating high calcium counts of about 2250 at 3.8 keV in Fig. 6 (b). Calcium, in the presence of oxygen, reacts and forms calcium oxide. Calcium oxide reacts efficiently with water, forming Portlandite (Ca(OH)_2_). Portlandite influences the cement’s hydration, resulting in increased ettringite production and a denser microstructure in the cured paste. This explains why the concrete’s strength is higher^84^. Thus, Fig. 6(a) indicates a compact structure and fewer voids among constituents of concrete, improving the cement matrix’s bond with the aggregate particles and promoting aggregate interlock, which in turn increases the concrete’s compressive strength^24^. Portland cement hydrates produce C-S-H. This is primarily responsible for the strength of cement-based materials. In most concrete, they are the primary binding agent or the glue^85^. This indicates that the hydration of binders is not solely determined by the hybrid fly ash with OPC activity index without considering the contents of active microstructural compositions of aggregates.

**Fig. 7:**
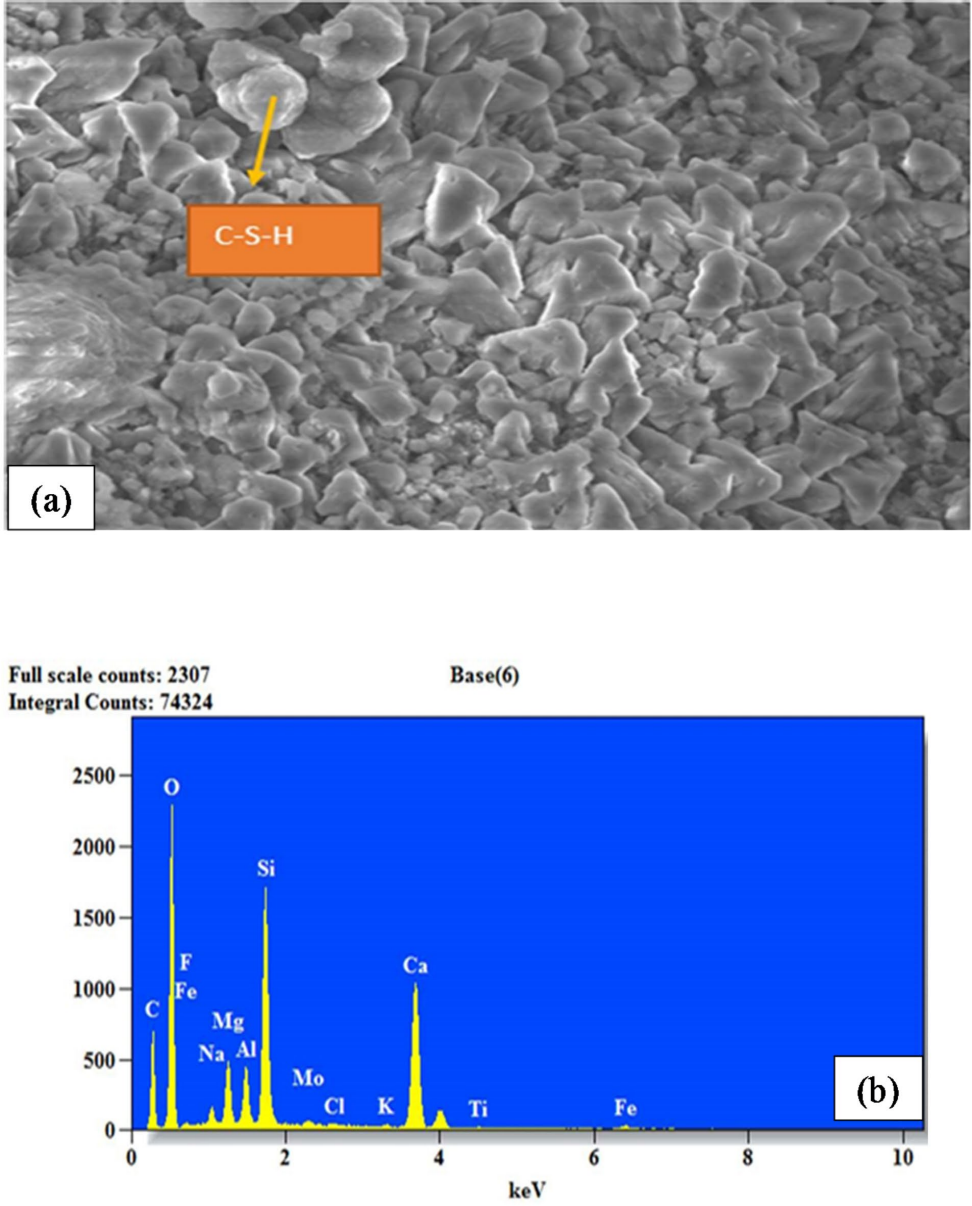
Microstructural analysis of concrete (**a**) SEM micrograph for 10%M sand and 10%fly ash (**b**) EDS for 10%M sand and 10%fly ash.

Compared to the result in Fig. 6 (a), a similar micrograph was observed in Fig. 7 (a) for the M sand-fly ash-based concrete sample. Figure 7 (a) illustrates a compact structure and fewer voids. As shown in Fig. 7 (b), silicon exhibited the highest element in the mix, with approximately 1750 counts at 2.8 keV. This was followed by calcium, with about 1200 counts at 3.8 keV. Thus, silicon and calcium, in the presence of oxygen, generate silica (SiO_2_) and quicklime (CaO). Calcium oxide primarily contributes to initial strength through hydration reactions, while silicon oxide enhances long-term strength and durability by forming C-S-H and pozzolanic activity. Balancing these components is crucial for optimizing the mechanical properties and performance of concrete. Besides, CaO and SiO_2_ contribute to a denser and more cohesive microstructure in concrete, as evident in Fig. 6(a), leading to better compressive strength. Adding fly ash to the mixture is responsible for the blend’s highest silicon level. This is because fly ash is mainly composed of silica (60–65%), alumina (25–30%), and magnetite (6–15%) in its overall chemical composition^86^. Therefore, secondary hydration products can be formed when pozzolan (in this case, fly ash) and calcium hydroxide from cement hydration react. The active silica (SiO_2_) and alumina (Al_2_O_3_) components in the mix react with calcium hydroxide and generate secondary reaction products, such as C–S–H and C-A-H^87^. These secondary hydration products enhance the performance of the cementitious composites, contributing to the strong cementitious matrix and resulting in strength development^88^.

### ML models

#### Hyperparameters of the ML models

The architecture, functions, and hyperparameters were optimized during the training phase to enhance model performance. To improve prediction accuracy, we employed a rigorous trial-and-error process. For the XGBoost model, the hyperparameters were set to n_estimators = 105, learning rate = 0.3, and max_depth = 14. In the case of the LSTM model, the hidden node count was set to 60, with the sigmoid activation function applied, 450 epochs, and a batch size of 12, using the Adam optimizer for optimization. For the Gaussian Process Regression (GPR) model, the radial basis function width was set to 0.30, and the Gaussian noise was adjusted to 0.03%. These hyperparameters were carefully selected through experimentation to ensure accurate prediction of compressive strength. In addition, the hyperparameters were continuously adjusted based on model performance during training, with cross-validation techniques employed to prevent overfitting and ensure generalization. Performance metrics, such as RMSE and R², were monitored throughout the process to evaluate the impact of different hyperparameter settings on the model’s prediction accuracy.

### Statistical results and regression plots

Detailed analyses of the four suggested models formed the basis of our research. Tables 4 and 5 provide a comprehensive analysis of crucial statistical parameters in both testing and training. Table 5 offers concise insights into the effectiveness of each model through comprehensive score analyses. There were apparent differences between the four models during training and testing. The XGBoost model achieved outstanding performance during training and testing, with an impressive R^2^ of 0.99999. However, the LSTM model achieved an R^2^ of 0.93456 during training and 0.93269 during testing. GPR achieved an R^2^ of 0.98493 and an RMSE of 1.51029 during training, closely following the performance of XGBoost. Nevertheless, its performance weakened during testing, as shown in Table 5, as demonstrated by a decrease in predictive precision. Testing revealed that the SVM model performed well, which achieved R^2^ values of 0.96757 and RMSE of 2.32183. Testing of the SVM model resulted in a score of 24. The performance indicators provided in Tables 4 and 5 complement the comprehensive evaluation and highlight the significance of systematically analyzing models to identify the optimal model for forecasting the strength of concrete.

**Table 4:** Performance metrics of the ML model in training.

Indicator	XGBoost	LSTM	SVM	GPR
R^2^	0.99999	0.93456	0.97989	0.98493
Score	4	1	2	3
WMAPE	0.00024	0.10455	0.05406	0.04094
Score	4	1	2	3
NS	0.99999	0.93292	0.97949	0.98480
Score	4	1	2	3
RMSE	0.04378	3.17274	1.75453	1.51029
Score	4	1	2	3
VAF	99.99873	93.40169	97.97564	98.48866
Score	4	1	2	3
LMI	0.99946	0.76340	0.87767	0.90734
Score	4	1	2	3
RSR	0.00357	0.25900	0.14323	0.12329
Score	4	1	2	3
MAE	0.00550	2.43049	1.25667	0.95182
Score	4	1	2	3
Total Score	32	8	16	24

**Table 5:** Prediction performance of the ML model in testing.

Indicator	XGBoost	LSTM	SVM	GPR
R^2^	0.99639	0.93269	0.96757	0.95938
Score	4	1	3	2
WMAPE	0.02382	0.11286	0.08051	0.08175
Score	4	1	3	2
NS	0.99633	0.92941	0.96516	0.95717
Score	4	1	3	2
RMSE	0.75317	3.30468	2.32183	2.57408
Score	4	1	3	2
VAF	99.63786	93.14902	96.74315	95.93472
Score	4	1	3	2
LMI	0.94915	0.75908	0.82814	0.82549
Score	4	1	3	2
RSR	0.06055	0.26568	0.18666	0.20694
Score	4	1	3	2
MAE	0.53324	2.52638	1.80213	1.82999
Score	4	1	3	2
Total Score	32	8	24	16

**Fig. 8:**
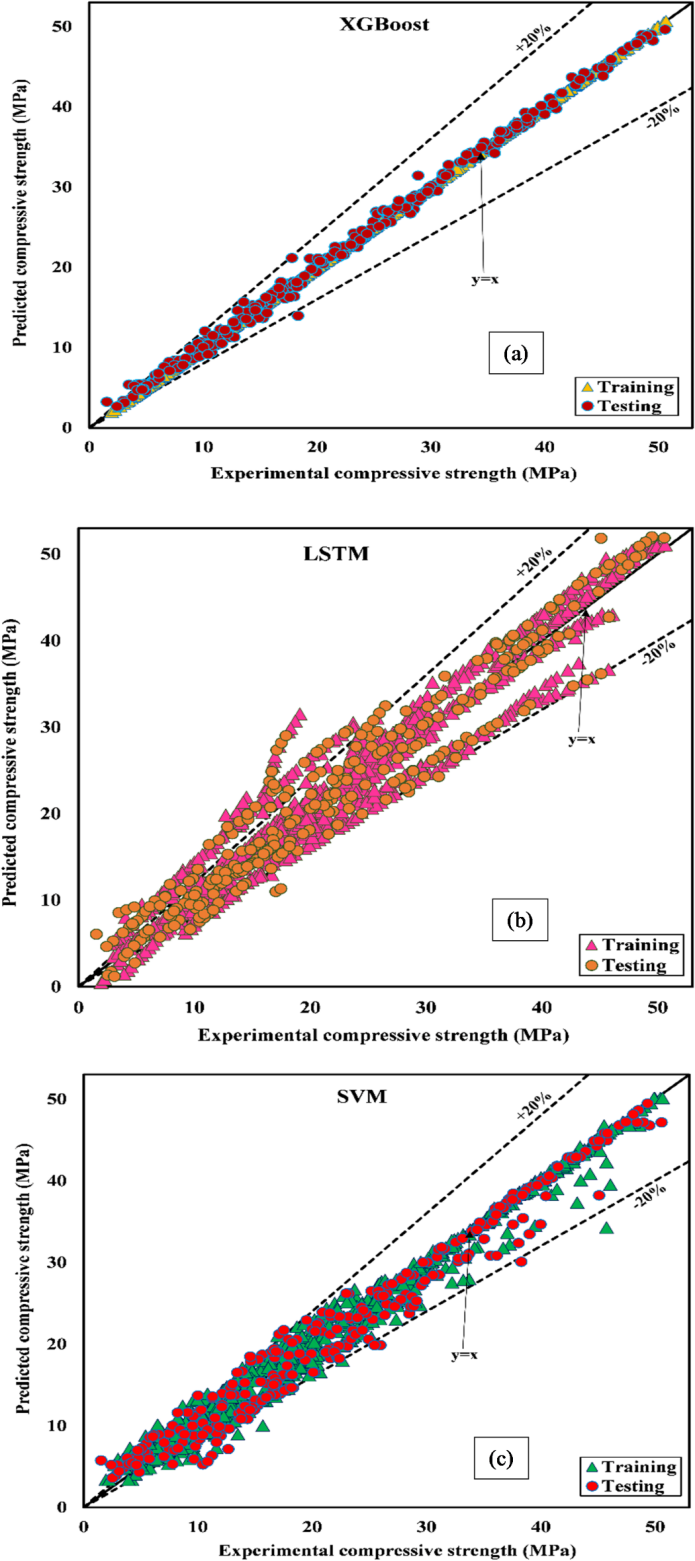

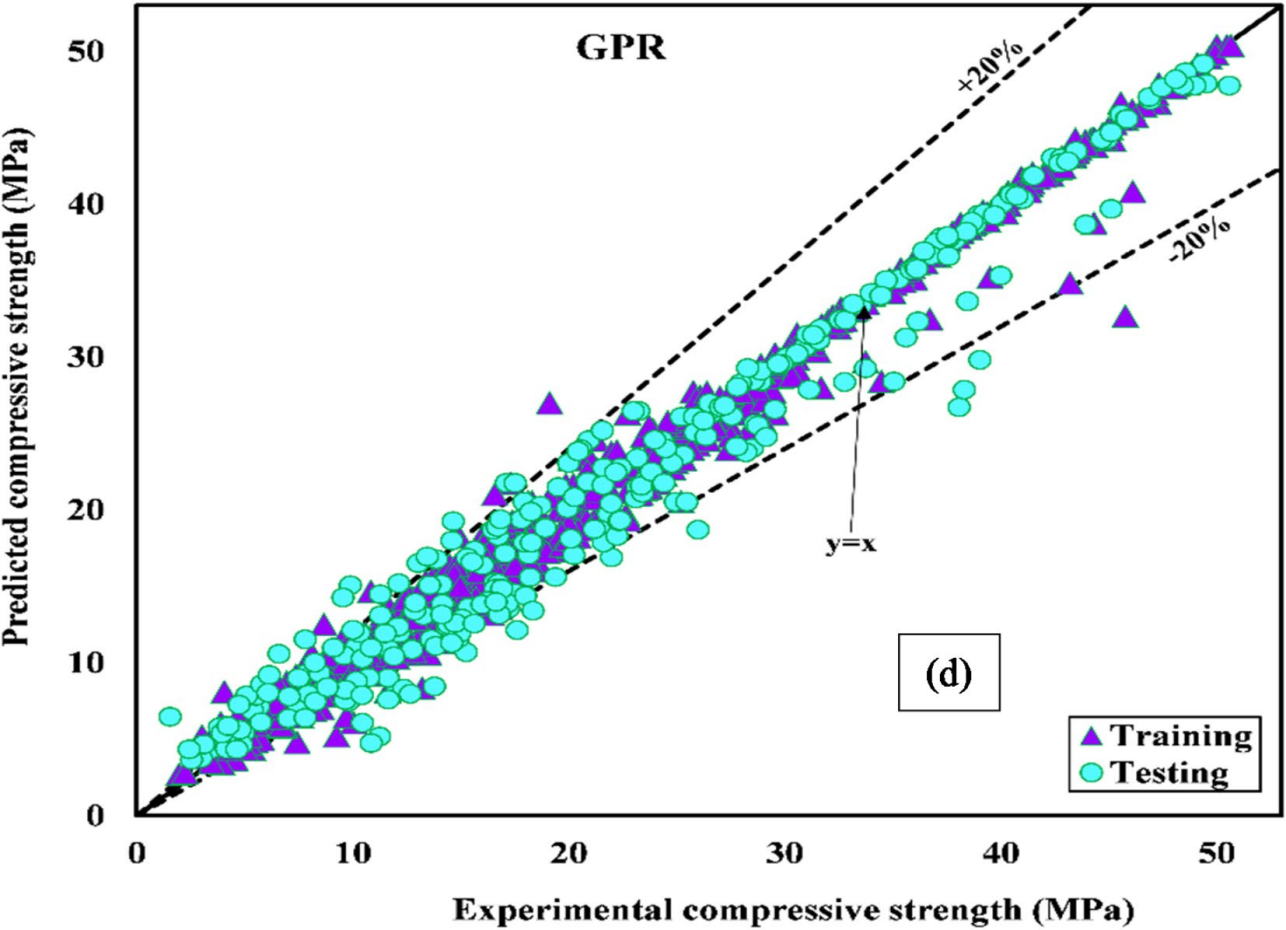
Regression plots for (**a**) XGBoost, (**b**) LSTM, (**c**) SVM, and (**d**) GPR models.

A regression plot showing the performance of XGBoost, LSTM, SVM, and GPR in predicting concrete strength is shown in Fig. 8 (a)-(d). For a model to be considered best, the data points must align with perfect prediction y = x. Figure 8 (a) and (d) show that XGBoost and GPR performed best during training. The predicted and observed compression strength values were close (y = x). In contrast, the LSTM model exhibits a distinct dispersion of data points around the line (y = x), as shown in Fig. 8 (b). A deviation from the line y = x can be seen in Fig. 8 (c) for the predicted SVM data point.

During the testing stage, the performance of XGBoost in Fig. 8 (a) remained unchanged, while that of GPR in Fig. 8 (d) decreased. The SVM model in Fig. 8 (c) outperformed GPR in Fig. 8 (d), with the line of regression perfectly aligned with y = x. According to Fig. 8 (b), the data points for the tested LSTM model are significantly separated from the line y = x, indicating that the model performs poorly. XGBoost achieved a higher R^2^ value during training and testing, demonstrating higher predictive accuracy. These findings showed that a comprehensive evaluation of various ML algorithms should be used in determining the most accurate model for predicting the compression strength of concrete.

### Regression error characteristic (REC) curve

**Fig. 9:**
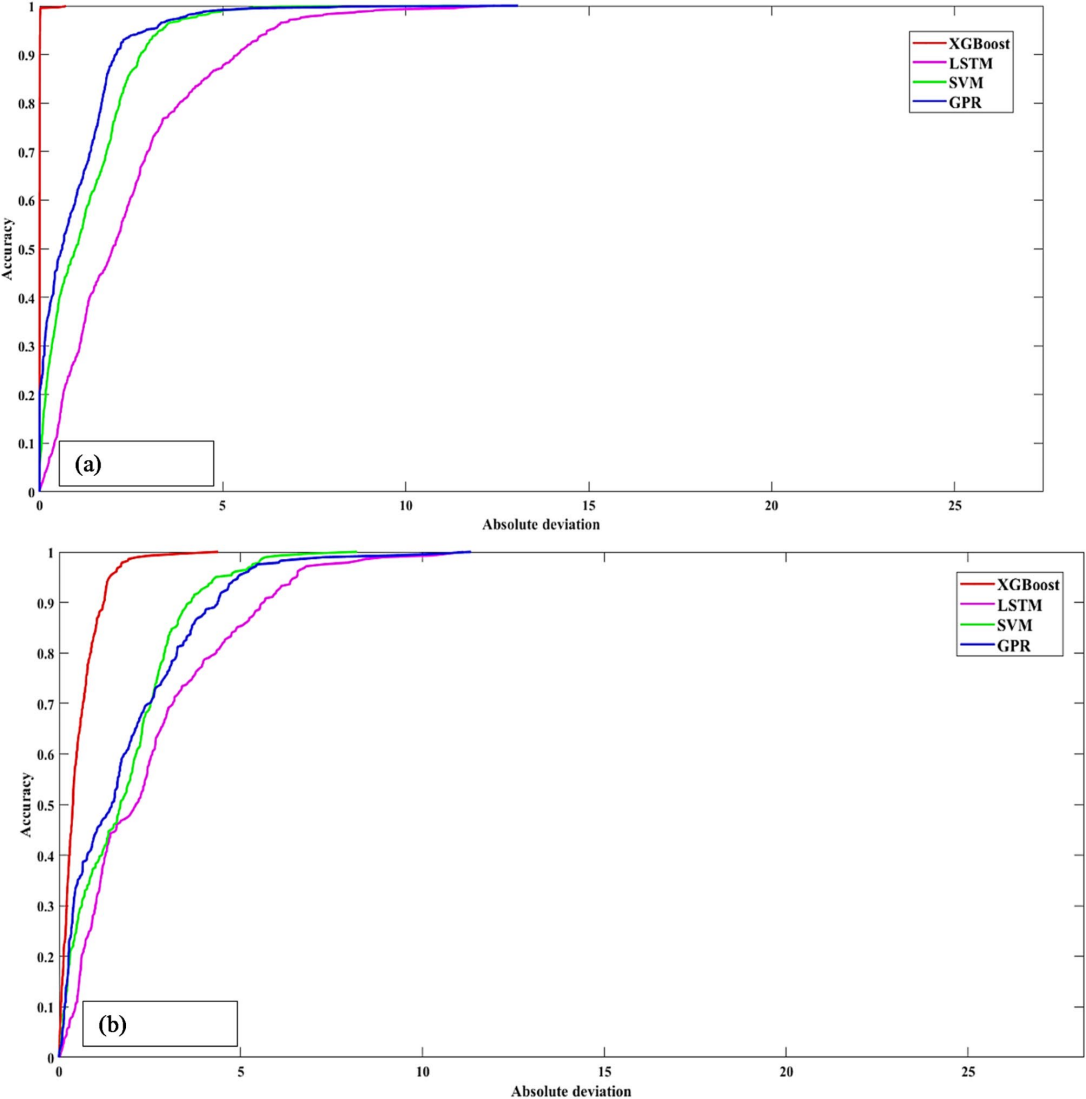
REC curves in (**a**) the training stage and (**b**) the testing stage.

To obtain a more comprehensive understanding of the dynamic relationship between error tolerance and prediction accuracy, the REC curve is an extremely useful visualization tool^89,90^. This curve provides a concise representation of the accuracy and reliability of a model by plotting the error tolerance against the percentage of predicted values that fall within that tolerance. Regarding this instance, the x- and y-axes represent the accuracy and error tolerance of the regression function, respectively. When a model is close to the top-left corner of the graph, it indicates accuracy. A lower area over the REC curve (AOC) suggests that the model is performing exceptionally well and provides a precise measurement of the model’s capabilities. The REC curves for both the training and testing phases are displayed in Fig. 9 (a) and (b), which provide a concise overview of the predictive capabilities of the models.

**Fig. 10:**
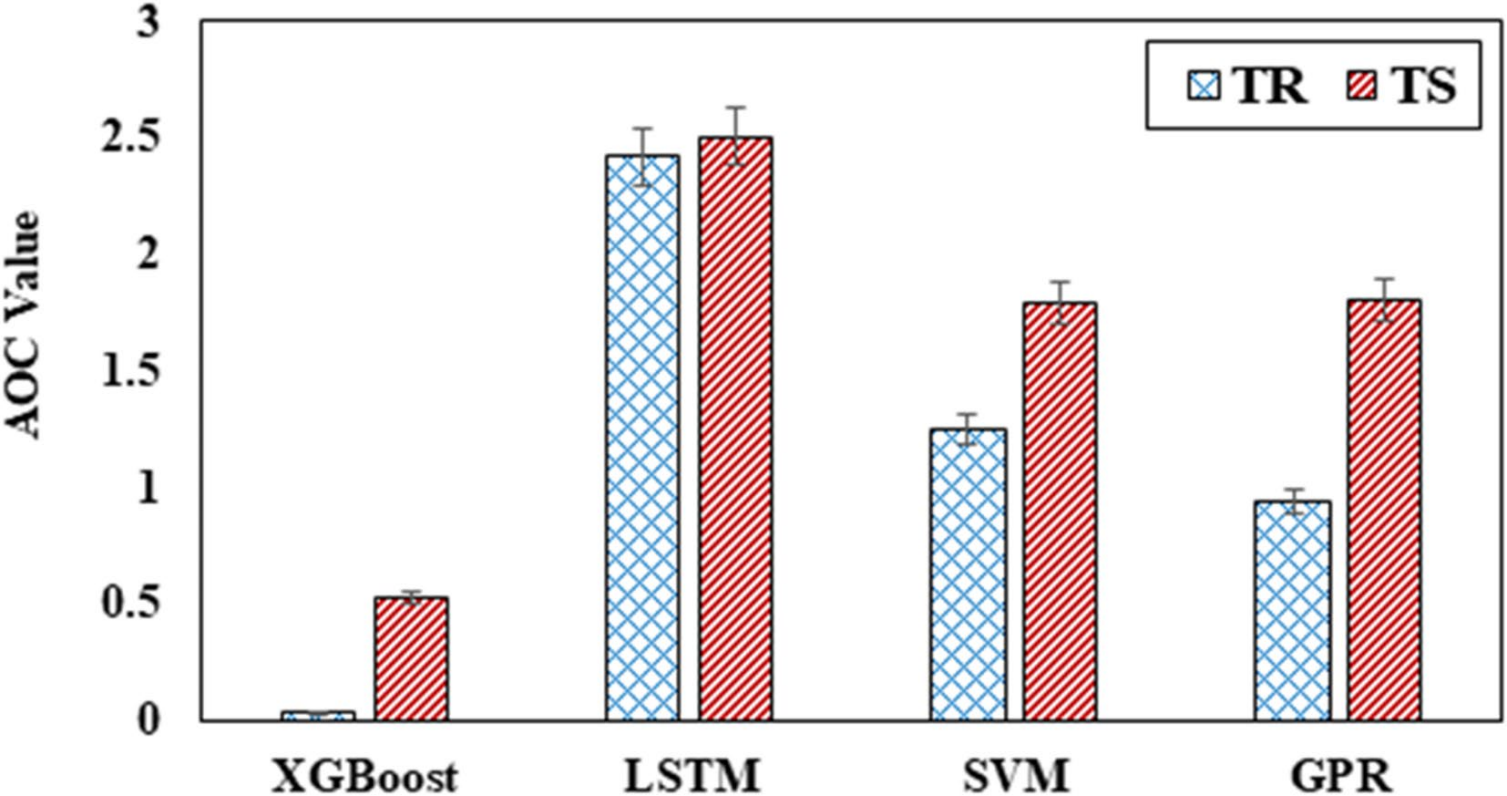
AOC values in the training and testing stages.

An insightful visual analysis revealed that the LSTM model has a relatively low level of accuracy during both the training and testing phases (Fig. 9 (a) and (b)). This is evident from the distance its curve is from the upper left corner of the graph. In contrast, the XGBoost model demonstrated a predictive accuracy that is extremely close to perfect. Its curve overlaps with the upper left corner in Fig. 9 (a) and is near this corner in Fig. 9 (b). Investigating the AOC values of the models, which are shown in Fig. 10, is another step in the process of comparing the performances of the models. Because the XGBoost model has the lowest AOC during both training (AOC = 0.0359) and testing (AOC = 0.5255), it is the model that performs the best in terms of predicting compression strength. On the other hand, the LSTM model consistently achieves higher AOC values throughout the training and testing phases. Considering this, the REC curve and the AOC values further prove that the XGBoost model possesses superior predictive capabilities shown in Fig. 10. These findings agree with the conclusions drawn from the statistical analysis and regression graphics. The results of this study provide evidence that the model is effective and highlight the potential of the model to make accurate predictions regarding the concrete’s strength.

#### Uncertainty analysis

Uncertainty analysis is a method for evaluating possible mistakes and unknowns in prediction models. There are multiple steps in an uncertainty analysis process. Uncertainty must be identified in the first stage. We need to put a number on the uncertainty associated with each source. The computation of standard deviations ($\:{S}_{e}$) and mean error ($\:\widehat{e}$) may be needed, as demonstrated in Eqs. 24 and 25, for this purpose. The mean error of each parameter value, $\:{e}_{i}$, can be determined using Eq. 26, where $\:{d}_{i}$ is the actual value and $\:{y}_{i}$ is the predicted value of the output.

24 $$\:{S}_{e}=\sqrt{\frac{{\sum\:}_{i=1}^{n}({e}_{i}-\widehat{e})}{n-1}}$$

25 $$\:\widehat{e}=\frac{{\sum\:}_{i=1}^{n}{e}_{i}}{n-1}$$

26 $$\:{e}_{i}={y}_{i}-{d}_{i}$$

**Fig. 11:**
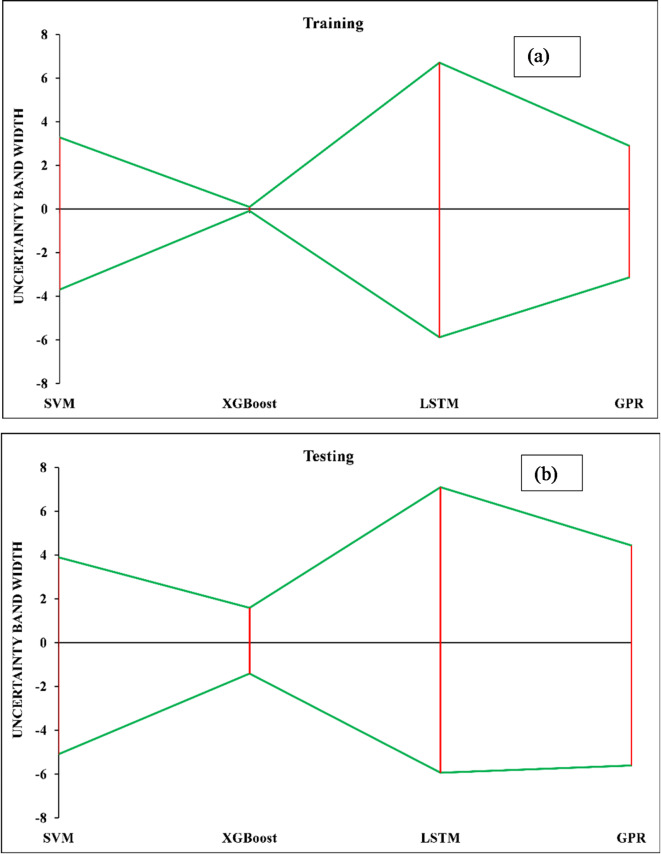
Sensitivity analysis for (**a**) the training set and (**b**) the testing set.

The methodology is implemented in three key steps: (1) combining individual uncertainties into a single measure to determine total uncertainty; (2) effectively communicating this uncertainty to guarantee accurate data interpretation; and (3) incorporating uncertainty analysis into scientific research to verify accuracy. Confidence intervals for the mean error are created using the Wilson score method by utilizing the estimated standard deviation ($\:{S}_{e}$) and mean error ($\:\widehat{e}$) values^91^. Table 6 presents the uncertainty analysis results for the training and testing phases clearly and concisely, providing a thorough overview of model performance. Additionally, Fig. 11 (a) and (b) provide a clear illustration of the uncertainty bandwidth comparisons between all models at these stages. Surprisingly, the XGBoost model performs best, as shown in Table 6, with the lowest mean error in both prediction phases. The GPR, SVM, and LSTM models trail during training; in testing, the order switches to SVM, GPR, and LSTM. Characterized by narrower uncertainty bandwidths, models with higher predictive power are identified. Visual proof of XGBoost’s narrower uncertainty bandwidth relative to the GPR, SVM, and LSTM models is shown in Fig. 11 (a) and (b), highlighting the model’s superior performance during training and testing. These empirical data emphasize the value of XGBoost in uncertainty analysis for rigorous scientific research and reinforce its status as a mainstay of predictive accuracy.

**Table 6:** Uncertainty results for the training and testing stages.

Model	Phase	Mean prediction error	Standard deviation of predicted error	Uncertainty bandwidth	95% prediction error	
XGBoost	Training	0.002868	0.043723	0.087446	−0.08458	0.090314
	Testing	0.088157	0.749318	1.498636	−1.41048	1.586793
LSTM	Training	0.413984	3.148007	6.296014	−5.88203	6.709998
	Testing	0.580113	3.259147	6.518294	−5.93818	7.098407
SVM	Training	−0.20642	1.743667	3.487334	−3.69375	3.280913
	Testing	−0.59937	2.247122	4.494245	−5.09361	3.894874
GPR	Training	−0.12071	1.506608	3.013216	−3.13392	2.89251
	Testing	−0.58758	2.510573	5.021146	−5.60873	4.433566

### Taylor diagram

**Fig. 12:**
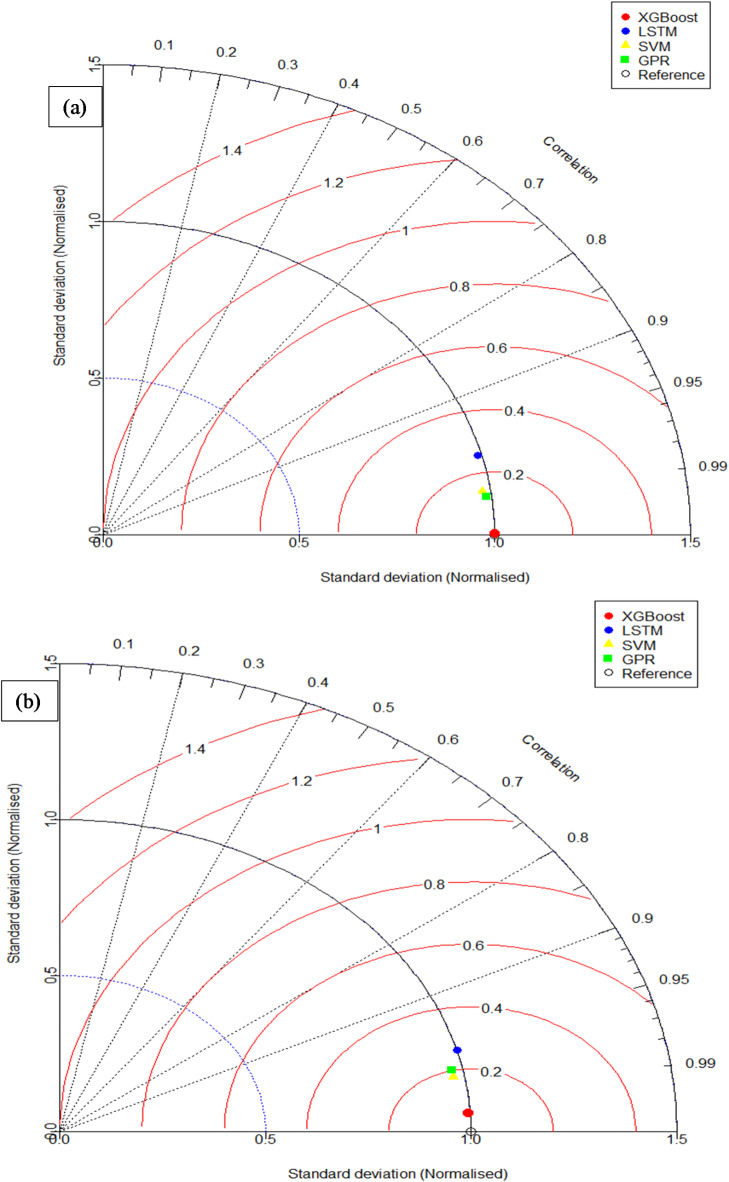
Taylor diagrams for (**a**) the training stage and (**b**) the testing stage.

Figure 12(a) and (b) show a detailed evaluation of the models’ performance during testing and training using the illuminating Taylor diagram^74,90^. By providing a holistic view of the models’ capacity to foretell the desired result, this visual representation is an essential tool for evaluating their predictive power. To quantify the performance of each model, our study utilizes three separate statistical measures: RMSE, standard deviation ratio (SDR), and R. The RMSE or distance from the measured point is the central reference point around which the Taylor diagram revolves. The reference model’s R and SD are set to 1 in this setting. The training phase for the output compression strength is illustrated in Fig. 12 (a), while the testing phase is depicted in Fig. 12 (b).

The correlation coefficient and standard deviation values of the XGBoost model were very close to those of the reference point, indicating an impressive level of agreement. In contrast, the LSTM model seems to stray slightly from the reference point during training and testing. A comparison of the overall accuracy of the proposed models in predicting compression strength reveals an overall story. The XGBoost model stands out as the pinnacle of accurate prediction, with its closeness to the “reference point” signifying its outstanding performance. The LSTM model, on the other hand, performs the worst because it has the lowest correlation in both prediction phases. The XGBoost model stands out for its exceptional accuracy and reliability in predicting compression strength, as the Taylor diagram shows, which summarizes a thorough evaluation of the model’s performance.

When compared to FTENN’s RMSE-focused evaluation in the study of Gupta and Kumar^11^, the XGBoost model in this study performed better in terms of predictive accuracy, obtaining R^2^ values of 0.9999 (training) and 0.9964 (testing). This study tackles broader sustainability by integrating fly ash and M sand, providing a scalable solution for replacing OPC and R sand, in contrast to FTENN’s focus on jarosite-mixed concrete. Furthermore, the best material ratios (25% fly ash, 50% M sand) and sophisticated validation procedures guarantee exceptional performance and usefulness for sustainable M40-grade concrete.

## Conclusions

This research replaced OPC and R sand with 0–85 wt% of fly ash and 0–100 wt% of M sand. The concrete mix proportions were designed using M40 grade. The concrete samples were tested for compressive strength after 3–90 days of curing. Furthermore, ML techniques were engaged to predict the compressive strength of the concrete samples using XGBoost, LSTM, SVM, and GPR models. Besides, the microstructures and elemental compositions of the concrete samples were characterized using SEM and EDS. Based on the experimental studies, the following conclusions can be drawn:

Replacing OPC and R sand with fly ash and M sand improves the compressive strength of concrete. However, the optimum compressive strength is attained at 25 and 50 wt% replacement levels for fly ash and M sand. After extensive learning, the XGBoost model proved to have the highest predictive power for forecasting the compressive strength in training and testing phases with 0.9999 and 0.9964 R^2^ compared to LSTM, SVM, and GPR with 0.9346 and 0.9327 R^2^, 0.9799 and 0.9676 R^2^, and 0.9849 and 0.9594 R^2^. A thorough validation process through the REC curve, uncertainty analysis, and Taylor diagrams proved that its accuracy outperformed that of LSTM, SVM, and GPR. SEM-EDS analyses revealed compact formations with high calcium and silicon counts in concrete samples containing M sand and fly ash, generating C-S-H for improved strength.

From this experimental investigation, the blended concrete obtained with 25 wt% replacement of OPC by fly ash and 50 wt% replacement of R sand by M sand can be used for structural applications. Utilizing fly ash and M sand helps manage waste, reduce CO_2_ emissions, and promote sustainable building and construction practices. As a result, it helps mitigate the problems associated with pollution and disposal.

### Limitations and future recommendations

Despite its thoroughness, this study has a number of limitations that should be noted. First, only a limited number of replacement levels were available for OPC and R sand with fly ash and M sand, which might not adequately represent the possible performance of concrete at different replacement levels. Second, the size of the dataset used to train the machine learning (ML) models was limited, which might have an impact on how broadly the predictions can be applied to various concrete mix designs or environmental circumstances. Additionally, fly ash quality variability is a major problem because its characteristics vary depending on the coal type and thermal power plant operating conditions, which results in inconsistent material performance. Furthermore, localized variations within the samples might not be considered by assumptions made during the SEM-EDS characterization, such as the uniformity of microstructural compositions.

At high replacement levels of fly ash and M sand, it was found that hydration and strength development were adversely affected, requiring longer curing times to reach the required concrete strength. The performance of fly ash-M sand-based concrete is influenced by numerous factors, all of which are challenging to include in a single explicit model, thereby restricting the findings’ applicability. Furthermore, even though machine learning models like XGBoost show remarkable accuracy, there are still several issues with their dependence on experimental datasets, the requirement for intensive preprocessing, and their limited ability to apply to different concrete compositions without further training.

The absence of a thorough comparison between the suggested concrete mix (25 weight% fly ash and 50 weight% M sand) and traditional M40 concrete in terms of price, material availability, and regulatory compliance is another crucial factor. Understanding the findings’ wider applicability and practical ramifications requires this kind of comparison. Future studies should assess the suggested mix’s economic viability while accounting for regional availability, material costs, and logistics of transportation. Additionally, lifecycle assessments should be used to quantify the mix’s environmental benefits, such as lower carbon emissions and better waste management.

Future studies should concentrate on optimizing mix designs by looking into wider replacement ranges and enhancing material consistency in order to overcome these constraints. To improve the precision and generalizability of ML-based predictions, advanced predictive technologies like deep neural networks and hybrid models ought to be investigated. Explainable models should pinpoint the main elements affecting concrete strength, and data augmentation and transfer learning can be used to get around dataset constraints. For broader applicability, validation under various environmental conditions and mix proportions is essential.

Assessments of durability using carbonation, rapid chloride penetration, and sorptivity testing could be part of future research. The blended composition should also be used to test the performance of special concretes, such as fiber-reinforced, self-compacting, and lightweight concrete. Further understanding of structural integrity and performance would be possible through studies on thermal characteristics and non-destructive testing techniques. In order to support sustainable building practices, machine learning applications should be extended to include lifespan, lifecycle cost, and other long-term property predictions. Lastly, phase transitions and mineralogical alterations in the concrete matrix should be examined using X-ray diffraction analyses.

## Data Availability

All experimental datasets are publicly available via open access in the Zenodo Repository at https://zenodo.org/doi/10.5281/zenodo.13000483.
